# Translating neonatal microbiome science into commercial innovation: metabolism of human milk oligosaccharides as a basis for probiotic efficacy in breast-fed infants

**DOI:** 10.1080/19490976.2023.2192458

**Published:** 2023-04-03

**Authors:** David A. Mills, J. Bruce German, Carlito B. Lebrilla, Mark A. Underwood

**Affiliations:** a Department of Food Science and Technology, University of California-Davis, Davis, CA, United States; b Department of Viticulture and Enology, University of California-Davis, Davis, CA, United States; c Foods for Health Institute, University of California-Davis, Davis, CA, United States; d Department of Chemistry, University of California-Davis, Davis, CA, United States; e Department of Biochemistry and Molecular Medicine, University of California-Davis, Davis, CA, United States; f Division of Neonatology, Department of Pediatrics, University of California-Davis, Sacramento, CA, United States

**Keywords:** Gut microbiota, human milk oligosaccharides, *Bifidobacterium*, probiotics, premature infants, neonatal intensive care unit

## Abstract

For over a century, physicians have witnessed a common enrichment of bifidobacteria in the feces of breast-fed infants that was readily associated with infant health status. Recent advances in bacterial genomics, metagenomics, and glycomics have helped explain the nature of this unique enrichment and enabled the tailored use of probiotic supplementation to restore missing bifidobacterial functions in at-risk infants. This review documents a 20-year span of discoveries that set the stage for the current use of human milk oligosaccharide-consuming bifidobacteria to beneficially colonize, modulate, and protect the intestines of at-risk, human milk-fed, neonates. This review also presents a model for probiotic applications wherein bifidobacterial *functions*, in the form of colonization and HMO-related catabolic activity *in situ*, represent measurable metabolic outcomes by which probiotic efficacy can be scored toward improving infant health.

Consider the fragility of a typical human neonate. To say the infant is born naïve is a stark understatement. Humans are born with immature senses, nascent cognitive abilities, little muscle coordination, an undeveloped immune system, and limited ability to garner food more than reflexive behaviors (sucking, rooting, etc.). Consequently, early postpartum life for humans necessitates a bidirectional interaction within the mother-infant dyad to ensure a stable life trajectory for the infant. Given the overwhelming needs of the neonate *ex utero*, this stage truly represents a “fourth trimester,” a term coined by anthropologist Sheila Kitzinger^1^.

There is no question that this bidirectional interaction between mother and infant results in a bewildering number of new experiences for the infant; chief among them is the paradigm shift from predominantly intravenous nutrition facilitated by the placenta to enteral nutrition, produced by the mother in the form of breast milk, a fluid shaped by millions of years of mammalian evolution. While significant emphasis has been placed upon the role of milk in nourishment, evidence from various perspectives argues that acute protection of the infant from infection and inflammation is an equally compelling role for milk. The *in utero* environment is both supportive and protective, especially from microbial pathogens. Breaches in the microbial barrier to the infant’s environment are typically devastating to a pregnancy. At a relatively early stage of their development, human infants are born, dropped, figuratively and occasionally literally, into the mud. Their world is transformed, from a sterile environment to an environment teeming with microbes. It is this potentially catastrophic environmental transition that has placed a profound selective pressure on the genetics of lactation and the composition and functions of milk throughout mammalian evolution. The most recent investigations of milk support the hypothesis that milk is indeed far more than simple nourishment of the infant, it is a living, active, dynamic biological system sculpted by the evolutionary process to protect the infant from infection and guide the development of both the infant’s innate and adaptive immune systems and the developing intestinal microbiome. It has taken scientists considerable time and resources to discover that human milk is so broadly protective with the parallel emergence of two very different strategies. First, milk delivers antimicrobial proteins, peptides, lipids and enzymes as an awesome array of tactical weapons to protect the infant from pathogenic organisms. Second, milk components shape a symbiotic protective microbiome that influences and interacts with the developing immune system. What mothers have done is truly astonishing: recruit another kingdom of biology to protect their infants. This strategy poses a daunting tactical challenge for mothers: delivering in parallel antimicrobial components to the infant to ensure acute protection and milk components that support specific beneficial microbes within the infant. These components nourish not the infant but a unique bacterial population that provides metabolites and suppresses more inflammatory microbes, all with the selective advantage of protecting the infant!

## Early research on a breast milk-gut microbiome association

Since the early invention of the microscope by von Leeuwenhoek, scientists contemplated the utility of the bacteria they witnessed in feces. Early speculations by Frerich in 1846 declared “the bacteria neither aid nor interfere with the digestive processes” a view held by many at that time^2^. Between 1890 and 1930 three separate lines of research emerged that suggested there was something unusual about the gut microbiome of breast-fed infants. The first was microscopic observations by Henry Tissier^3^ of fecal smears from healthy breast-fed infants, revealing a near monoculture of “bifid” or Y-shaped bacteria, then termed *Bacteria bifidus*, an observation repeated frequently throughout the early 1900s^2,4–6^. This “microbiome” observation differed dramatically from infants who consumed bovine milk, or fermented bovine milks, whose feces contained an assortment of bacterial shapes^2,5–7^. The second observation was that breast-fed infant feces were consistently more acidic (pH ~ 5) by comparison to that from bovine milk-fed infants which was also more variable (pH ~ 6–8)^8–12^. The third observation was an early correlation drawn between infants that lack *Bacteria bifidus-*dominated feces and the increased occurrence of infant gut-borne pathogens^3^. This led to speculation that human milk, unlike bovine milk, contains a specific component that encouraged growth of *Bacteria bifidus* and that its metabolism was protective of the infant intestinal environment^7^. In one sense, this was an early observation of a “microbiome” deficiency among a “dysbiotic” cohort when compared to that in a healthy population – a common strategy employed in comparative gut microbiome studies today.

If *Bacteria bifidus* were frequently observed in breastfed infant feces in the early 1900s, what components of breast milk were they consuming that were lacking in bovine milk? Solving this mystery took additional decades of research recently elaborated in an excellent historical review by Clemens Kunz^13^. In short, dissecting of both human and bovine milk revealed specific, non-protein components, responsible for the growth of this key bacterium, which, at that time, was termed *Lactobacillus bifidus* ^9,14^ (see Box 1). Gorgy and coworkers used growth of *L. bifidus* on different fractions of human milk as a guide to reveal a “bifidus” factor that was separable from larger glycoconjugates (like mucins) and from smaller sugars like lactose^15^. This fraction contained fucose, galactose, and N-acetyl-glucosamine constituents, components that were known associated with blood group glycans, an aligned and emerging scientific area at the time. Subsequent research in the years between 1950 and 2000 documented the remarkable abundance of milk oligosaccharides in human milk by comparison to other milks, notably bovine milk^16^, as well as isolation and characterization of individual human milk oligosaccharide (HMO) species such as fucosyllactose, lacto-N-tetraose, lacto-N-fucopentaose, and difucosyllactose, among others^13^.

Box 1. A taxonomic evolution from Bacteria bifidus to *Bifidobacterium longum subsp. infan-tis*.

Microbial taxonomy is ever-changing. While Tissier^3^ identified the predominant bifid-shaped bacteria in the feces of breastfed infants, it took the trained eye of the famous microbiologist Sigurd Orla-Jensen in 1924^180^ to give a name to these unusual Y-shaped bacteria to the genus, *Bifidobacterium*. Adoption of the new genus name was not universal, however, and seminal work by Gyorgy^14^ defined similar infant isolates as *Lactobacillus bifidus* (later determined to be *Bifidobacterium bifidum*). *Bifidobacterium infantis* was first defined as a species in 1963^181^, with the species name becoming official in 1973^61^. This designation held until 2008, when Mattarelli and colleagues^62^ revised the species by combining then *B.* *longum*, *B.* *infantis*, and *B.* *suis* into three *B.* *longum* subspecies – subsp. *longum*, subsp. *infantis* and subsp. *suis*. This designation still stands today confirmed by numerous pangenomic analysis of the clade^57,58^. Although the shortened term “*B.* *infantis*” is still routinely used in the scientific literature to refer to the subspecies, the official name is *Bifidobacterium longum* subsp. *infantis*. Despite the advances in molecular taxonomy, this subspecies has been incorrectly designated for various commercial probiotic products^170^.

As analytical methods to characterize and purify HMOs advanced from 1950 to 2000, little cognate advance in the understanding of the intestinal bifidobacteria that grow on them took place. During that same time, the subject of probiotics experienced a renaissance with the growth of research on lactic acid bacteria associated with food fermentations and gut health, however research on the interaction between HMOs and infant-borne bifidobacteria was lacking. In the mid 2000s Katayama and colleagues characterized extracellular fucosidase^17^ and lacto-n-biosidase^18^ from *Bifidobacterium bifidum* and *Bifidobacterium longum* subsp. *longum* (herein termed *BL. longum*). If HMOs were truly the “bifidus factor” responsible for enrichment of bifidobacteria in infants, did all bifidobacterial species grow equally? Early efforts by Ward^19,20^ and LoCascio^21,22^ clearly showed that only select bifidobacterial strains grow well (i.e. to a high cell density) on HMO as a sole carbon source. Growth rates differed among bifidobacterial species, however individual isolates of *Bifidobacterium longum* subsp. *infantis* (herein termed *BL. infantis*) emerged as the most consistently robust HMO consumers, suggesting it was a characteristic of the subspecies^22^.

Key to elucidating the relationship between HMOs and bifidobacteria were innovations in profiling of discrete HMO species driven by a series of advances in analytical chemistry techniques. The analytical challenge here is not readily appreciated. More than 300 HMO structures may be possible, although only about 100 are detectible from any individual mother’s milk. Unlike many other metabolites, HMOs contain many isomers, compounds with the same composition but different structures possessing identical masses (e.g. lacto-n-neotetraose vs. lacto-n-tetraose). Differentiating these isomers was the subject of years of challenging analytical chemistry, particularly as separating the similar structures requires new chromatographic techniques. Additionally, the large amount of lactose in milk confounded clinical studies as it swamped any carbohydrate-specific methods of detection. Furthermore, the presence of peptides and proteins diminished the effectiveness of sensitive analytical techniques such as mass spectrometry. Thus, rigorous isolation of HMO pools was necessary to eliminate contamination from lactose as well as from peptides and proteins. Enrichment of the HMOs was accomplished using porous graphitized carbon (PGC), which released lactose in the void volume and retained proteins in the matrix^23,24^. This method was highly scalable allowing further enrichment of HMOs in gram amounts for biological assays. Although this enrichment procedure is today taken for granted, it was key to determining the biological activity of HMOs as it rigorously removed lactose, which was a contaminant in many early experiments on the biological functions of HMOs.

Initially, matrix-assisted laser desorption/ionization (MALDI) Fourier transform ion cyclotron resonance mass spectrometry (FT ICR MS) was used for compositional profiling of HMOs^25,26^. This high-performance method for mass detection yielded compositions including the number of hexose, deoxyhexose (fucose), and sialic acid, but no stereochemical information. Coincidentally, the development of nanoflow methods elsewhere coincided with earlier HMO studies. Development of a microfluidic (Lab-in-a-chip) device that included a PGC enrichment column and PGC separation column allowing injection and separation of HMOs produced the most comprehensive method for separating and identifying HMO components^27,28^. Using this nanoLC chip with a PGC as stationary phase for chromatographic separation and quadrupole time-of-flight (QTOF) analyzers for mass detection, a comprehensive analysis of each individual component could be routinely performed of HMOs in various settings including mother’s milk^29–31^, urine^32^, feces,^32^ and even blood^33^.

**Figure 1.**
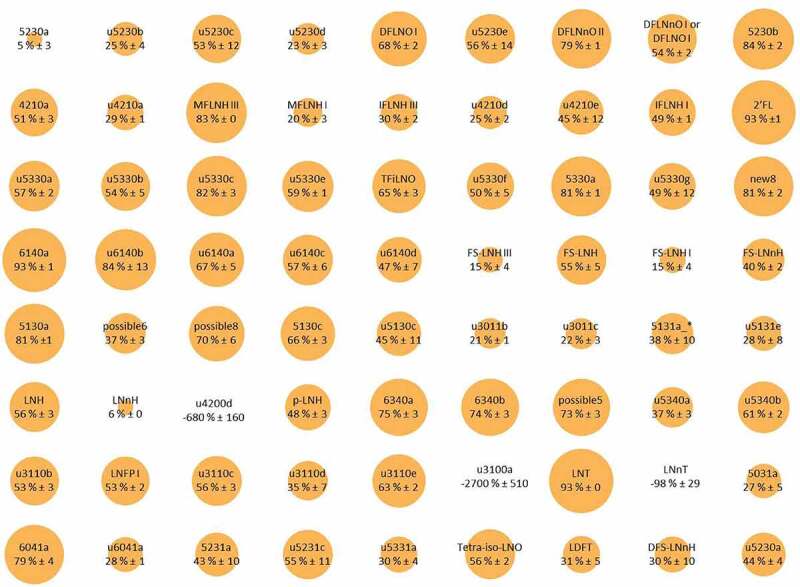
Bubble plot representation of simultaneous glycoprofiling of 78 different HMO structures differentially consumed by *BL. infantis*. The size of the bubble depicts the percent consumption of that specific HMO. Reproduced with permission from Strum et al.^42^ Copyright (2012) American Chemical Society.

Using purified HMO pools, these methods also allowed precise profiling of which specific HMO structures were metabolized by bacteria^34^. Strains of *BL. infantis* appeared to possess a generally similar HMO consumption, although some differences among strains were noted^22,35^. However, other bifidobacterial species isolates commonly found in the infant gut lacked this more robust phenotype. With the exception of *B. bifidum* ^36^, vigorous HMO consumption appears to be an episodic trait among isolates of *BL. longum* ^37^, *B. breve* ^38^, *B. pseudocatenulatum*, ^39,40^ and *B. kashiwanohense* ^41^ where most strains of those species do not grow on major components of HMOs, notably the fucosylated and sialylated HMOs. Among the *BL. longum*, *B. breve*, *B. kashiwnohense*, and *B. pseudocatenulatum* isolates that do robustly grow on HMOs, many consume only a portion of the HMO pool by comparison to *BL. infantis* ^42,43^ (Figure 1 depicts the consumption profile of *BL. infantis*). In general, this results in consumption of some fucosylated HMOs and non-fucosylated/non-sialyated HMOs but generally not the sialylated HMOs.

**Figure 2.**
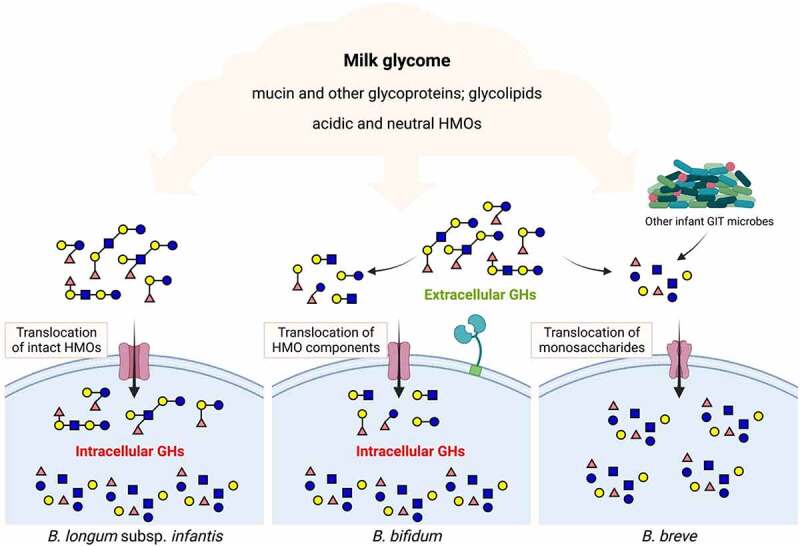
Prototypical strategies for HMO consumption by infant-borne bifidobacteria. GH, glycosyl hydrolase.

Early work on *B. bifidum* isolates showed consistently strong growth of HMOs, however it was noted that this species left degradation products of consumption, namely fucose and sialic acid, in the growth media suggesting extracellular hydrolysis of at least some HMOs^17,19^. Quite different consumption mechanisms elaborated by *BL. infantis* and *B. bifdum* illustrate the external (*B. bifidum*) versus transport and internal (*BL. infantis*) consumption mechanisms among bifidobacteria (Figure 2). Additional studies clearly showed the external degradation mechanism by *B. bifidum* enables cross feeding of HMO components of other bifidobacteria *in* *vitro*^44^. Given numerous genera present in the infant gut microbiome have been shown to externally consume HMOs^45,46^ like *B. bifidum*, it remains unclear what contribution these other clades might provide to HMO cross-feeding networks. Notably, others have demonstrated how mucin^47,48^ or HMO^49^ degradation can release sugar monomers that promote growth of enteric pathogens, suggesting a potential advantage to the host of transport and internal degradation of HMOs by *BL. infantis*.

**Figure 3.**
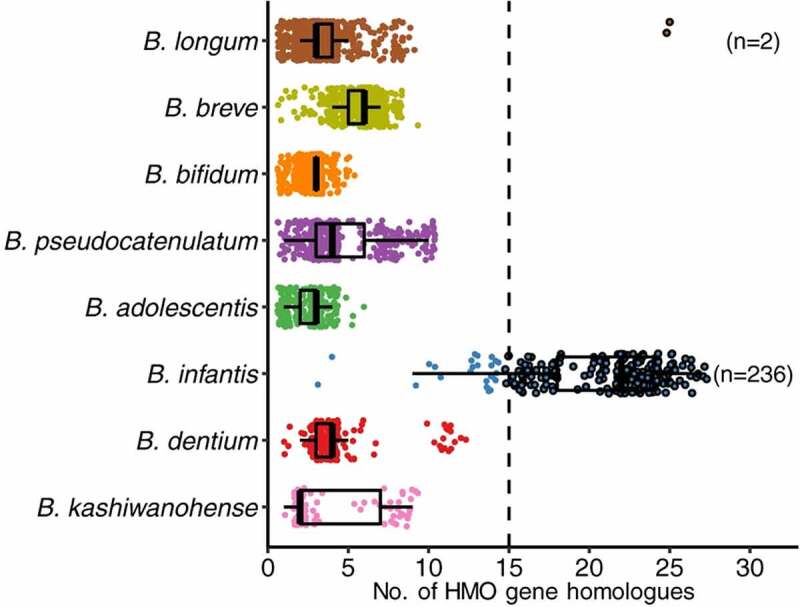
Pan-genomic demonstration of the abundance of HMO related genes in *BL. infantis* by comparison to other infant-borne bifidobacterial species. (Reproduced with permission from Zeng et al.^59^).

An early mechanistic explanation for the robust growth of *BL. infantis* on HMO emerged from the genome sequence first generated in 2008^50^. At the time, relatively few genes associated with specific HMO catabolism had been identified in any bifidobacterial strain. Within *BL. infantis* the bulk of the HMO consumption genes were co-localized within a single 43 Kb locus. This HMO cluster contained all glycosyl hydrolases needed to cleave the various HMO linkages, namely intracellular fucosidase^51^, sialidase^52^, β-galactosidase^53^, and N-acetyl-β-hexosaminidase^54^ activities along with a number of associated ABC transporters^55,56^. This suite of genes linked to HMO catabolism was confirmed in other *BL. infantis* isolates^35^ and subsequent pan-genomic analyses aligned with this view that the subspecies diverged from other *B. longum* subspecies with HMO consumption as a common phenotype^57–60^. The genomic work also explained the lack of arabinose utilization by *BL. infantis*, a long-known phenotype that separates it from other *B. longum* subspecies^61,62^. Interestingly, the remnants of arabinose utilization genes are present in *BL. infantis* however they are disrupted by fucosidase gene and associated permease, suggesting a unique specialization of this subspecies toward the breast milk niche and away from utilization of plant glycans^50^. A recent analysis of over 30,000 metagenome assembled genomes (MAGs) from the infant gut microbiome clearly shows the unique presence of genes within the 43 Kb HMO cluster among several hundred MAGs of *BL. infantis*, validating these original observations^59^ (Figure 3). These researchers also noted a higher relative abundance of *BL. infantis* among all *BL. longum* genomes in infant metagenomes worldwide, suggesting that large HMO cluster provides a competitive advantage for colonization of breast-fed infants. For more information on the different mechanistic pathways of HMO consumption by infant-borne bifidobacteria, readers are referred to an excellent review by Katayama and colleagues^63^.

## Are bifidobacteria a common presence in the breast-fed neonate gut or not? What’s old is new again

As insight emerged from early HMO-bifidobacterial work in the mid 2000s, a new technology came along that disrupted the field and put into question the importance of bifidobacteria in infants. Prior to the 2000s, the consensus view was that bifidobacteria routinely represent a predominant sector of the breast-fed infant gut microbiome, a view derived from generations of scientists employing microscopic, culture-based observations and first-generation non-culture-based methods^64^. Typically, this bifidobacterial predominance was observed after a short window early in lactation, wherein more aerobic taxa (i.e. enterococci, streptococci, staphylococci, *Escherichia/Shigella*) initially dominate^65,66^. However, in the mid 2000s, next-generation DNA sequencing provided a breathtaking new capacity to profile the assemblies of uncultured bacteria in the gut, thus providing unique insights into this ecosystem. In 2007, a seminal study by Pat Brown, David Relman, and colleagues at Stanford employed both 16S rRNA gene microarrays and sequencing of 16S rRNA gene amplicons to profile the microbiome of 14 Californian infants over a period of 1 year^67^. This was the first in-depth look at the “uncultured microbiome” in the developing infant. However, despite probing this dataset in various ways, they did not witness predominance, or even much of presence, of bifidobacteria. At the time, the authors noted:

“*Although it is conceivable that there are geographical or demographic differences in the prevalence of Bifidobacteria, we suspect that the emphasis on Bifidobacteria in studies and reviews of the infant GI microbiota may be out of proportion to its prevalence, abundance, and relevance to health*”.

As it turned out, numerous studies have since shown geographic differences in bifidobacterial populations among infant gut microbiomes around the world, and their presence is increasingly linked to infant health status^65,68–76^. In a sense, this early confusion by the first “omics-driven” survey of the infant gut microbiome is a cautionary tale for scientists using new approaches to describe any well-studied system. With this new technology comes the potential for new insights driving an appropriate and compelling desire to re-interpret existing dogma. However, insight through that new window might still be hazy, and in this case, the dogma bit back.

Prior to that seminal study, geographic differences among *BL. infantis* presence in infants were already a focus of attention. *BL. infantis* observed in Ghana cohorts was noted to be missing in New Zealand and United Kingdom cohorts^77^, and differential immunological responses to these bifidobacterial strains were correlated to incidence of atopy^64^. Once the robust HMO consumption capacity of *BL. infantis* was established via phenotypic and genotypic means, more researchers examined the geographic distribution of the subspecies among various infant cohorts^75,78–80^. Notably, *BL. infantis* appears less prominent in USA and European cohorts^70,81,82^ in comparison to Bangladeshi^83^, rural Malawi^84^, rural Venezuela,^84^ and Indonesian populations,^80^ suggesting a possible relationship to the level of industrialization experienced by a population. This geographic disparity was amplified in a recent survey by Sonnenburg and coworkers^85^ who compared infant metagenomes from the Hadza, a group of modern hunter-gatherers in sub-Saharan Africa, to aged-matched cohorts from Europe and the USA. These researchers also noted a high abundance of *BL. infantis*, and their HMO-utilization genes, among Hadza infants compared to infant cohorts from Europe or the USA. Fascinatingly, Jarvinen and coworkers recently identified a high population of *BL. infantis* within infants from rural Mennonite communities by comparison to a neighboring community in New York,^74^ indicating regional pockets of high *BL. infantis* exist within the United States.

Why might the “champion” HMO-consuming bifidobacterial subspecies^86^ be so differentially represented around the world? Sonnenburg and colleagues^85^ argued that *BL. infantis* overrepresentation among more rural regions is a reflection of its loss due to a myriad of factors associated with modern lifestyles in more industrialized regions. Indeed, the loss of intestinal taxa associated with industrialization is now the focus of efforts to preserve our “ancestral” microbes with the eventual goal of employing these strains in intestinal health applications^87,88^. It is easy to compile the various ways modern lifestyles might influence the early infant microbiome, such as use of antibiotics^89^, birth delivery mode,^90^ and introduction of formula or complementary feeding^91^ among others. Such changes may even be magnified across generations, i.e. the grandmother born by cesarean section and formula fed in the 1960s may not have received an optimum colonization from her mother, thus was unable to pass the microbes selected by evolution to her daughter and granddaughter even though each was born vaginally and breast-fed. Recently, Taft and colleagues^70^ postulated that the level of fecal bifidobacteria, and *BL. infantis* in particular, is associated with the regional history of breast-feeding practice wherein longer durations of breastfeeding by a population aligned with a higher level of *BL. infantis*.

## Innovation – the translation of *functional* probiotics into the neonatal intensive care unit

Examined in various surveys, the gut microbiome of premature infants stands in stark difference to that observed in healthy breastfed infants^92^. This is, perhaps, not surprising as the infant microbiome is shaped by early environmental exposures to microbes, which can be dramatically different in premature infants where mode of delivery, diet (i.e. breast milk or formula), use of antibiotics, respiratory support and use of proton pump inhibitors, among many other interventions, are factors that influence the early life gut microbiome^93^. In addition, essentially every aspect of the innate and adaptive immune systems of the preterm is immature, poorly regulated and dysfunctional including intestinal motility, acid production, apoptosis, tight junction composition, mucus production, secretion of antimicrobial peptides and mechanisms of regulation of inflammation altering not only the gut microbiome but the host responses thereto. In general, various surveys of premature infant gut microbiomes, both before and after the advent of next-generation sequencing microbiome analyses, have identified a lower overall diversity, increased relative abundance of Enterobacteriaceae and Enterococceae and decreased bifidobacterial populations as hallmarks of premature infant microbiome^94,95^. By comparison to a healthy bifidobacterial-dominant, term breastfed infant gut microbiota, the “dysbiotic” premature infant microbiota is likely to trigger higher levels of endotoxin, a weakened barrier function, bacterial translocation, and inflammation^96^. These changes appear to be central to the pathogenesis of necrotizing enterocolitis (NEC) and late-onset sepsis in preterm infants.

Even before the common use of the term “probiotics”, these differences led to proposals to colonize premature infants using “normal intestinal flora” in hopes of stabilizing their microbiome and preventing the onset of disease^97^. Of course, this was not a new concept as Tissier^7^ had discussed such an approach to treat infant disease more than 100 years ago. Early trials on probiotics in premature infants employed commercial strains of *Lactobacillus*, *Bifidobacterium*, or even the yeast *Saccharomyces*^98,99^. The most recent meta-analyses of prophylactic administration of probiotics in preterm infants include observational cohorts (30 studies, more than 77,000 preterm infants)^100^ and randomized controlled trials (56 trials, more than 10,000 preterm infants),^101^ and have demonstrated significant decreases in NEC, late-onset sepsis, and death in this highly vulnerable population. Routine prophylactic administration to preterm infants varies widely from high in Japan, Australia, New Zealand, Germany, and Scandinavia to moderate in Canada and low in the U.K. and U.S.^102^. Nearly all reviews cited the need for more information on strain specificity and mechanism of action as it is exceedingly difficult to compare probiotic trials of vastly different microbes. For example, it is likely that a mechanism of action of a probiotic yeast *Saccharomyces cerevisiae* (commonly called *Saccharomyces boulardii* although “*boulardii”* is not an accepted species name) is not the same as a probiotic *BL. infantis* given the microbes evolved within entirely different environments. Others argue that much more consideration on the safety of probiotics in fragile neonates is warranted^103^. A 2021 statement from the American Academy of Pediatrics echoed these concerns and cautioned against routine use of current commercial probiotics in premature infants citing lack of FDA-regulated, pharmaceutical-grade products and thus the potential for harm^104^, while the European Society for Pediatric Gastroenterology, Hepatology and Nutrition, the American Gastrointestinal Association and the World Health Organization examined the same data and provided conditional recommendations for routine probiotic use in preterm infants^105^. **Box 2** presents some of the regulatory challenges pertinent to the study and implementation of probiotics in the U.S.

Box 2.  Oversight of U.S. probiotic clinical trials and future clinical directions

In 2004, Underwood and colleagues sought direction from the U.S. Food and Drug Administration (FDA) regarding clinical trials of probiotics. At that time, the view of FDA was that a clinical trial could be performed without the oversight of the investigational new drug (IND) process if the primary outcome was not to prevent, treat, or mitigate a disease, but rather to alter the composition of the intestinal microbiota. The Institutional Review Board (IRB) at UC Davis agreed and approved the early trials^116,189–192;^ the National Institutes of Health also agreed with this approach and provided funding. Subsequently, the FDA changed their guidance, requiring IND oversight for clinical trials of probiotics in preterm infants regardless of the measured outcome. Given the hesitation of probiotic manufacturers to participate in the IND process, it has become more challenging to perform studies of probiotics in U.S. NICUs, though analysis of samples from NICUs outside the U.S.^193^ and retrospective cohort studies^117^ are still possible. A large multi-center clinical trial of a *Lactobacillus* probiotic with IND oversight is nearing completion (Clincaltrials.gov NCT03978000), however to date there are currently no U.S. clinical trials with IND oversight of an HMO-consuming probiotic.

Recent network meta-analyses have summarized efficacy in the prevention of NEC, death, and sepsis of several different probiotic products^93,97,187^. Unfortunately, most of the clinical trials included in these meta-analyses were relatively small studies comparing a single probiotic product to placebo in different locations with differing baseline incidences of the primary outcomes. To answer the question of which probiotic and dose are most likely to decrease the risk of NEC, death, or sepsis, the ideal studies would be large multicenter cluster-randomized crossover trials adequately powered to look at NEC as an outcome (e.g. multiple NICUs randomized to probiotic A vs. B for 1–2 years then crossed over to the other probiotic). Smaller trials could still be helpful if they included one or more functional outcomes as proposed herein for example, a clinical trial including 100–200 preterm infants randomized to probiotic A or B would not be adequately powered for NEC as a primary outcome but could determine which probiotic lowered fecal pH or increased fecal lactate, acetate, or indole lactic acid. In the absence of such trials, the clinicians must consider whether routine probiotic prophylaxis for very preterm infants is justified and if so which of many probiotics is most likely to improve clinical outcomes. To address the first question, we have advocated for including the parents in this important discussion^188^ and considering the baseline rates of NEC and late-onset sepsis in a given NICU and the risks associated with probiotic administration (including probiotic sepsis and the potential for contamination of commercial probiotics). To address the second question, key considerations include evidence of clinical benefit (has the product been shown to be efficacious?), evidence of good-manufacturing practices in all aspects of probiotic production and distribution (is the manufacturer able to demonstrate reliable purity and viability of the probiotic strain(s)?), capacity of the local microbiology lab to detect the administered probiotic in infant blood cultures, and insights into the most effective antibiotic treatment options for the rare cases of invasive probiotic sepsis (most bifidobacteria are sensitive to penicillin, ampicillin, vancomycin, and clindamycin but resistant to aminoglycosides and metronidazole)^194,195^. Development and validation of biomarkers of microbiome and host gut function as advocated in this review would be a significant step forward in identifying infants at highest risk and probiotics with the highest potential benefit. The NEC society is a nonprofit organization and has prepared a probiotic toolkit for clinicians interested in considering probiotic prophylaxis (NECSociety.org).

In addition to regulatory and safety concerns, key questions regarding prophylactic administration of probiotics to preterm infants include optimal strain or combination of strains, optimal dosing and timing of administration, and equally important, optimal performance criteria. What *function* does a probiotic need to perform to reliably benefit the neonate? Of equal importance, what diagnostics demonstrate that probiotics are performing their function(s)? To consider this we need to understand the ability of a microbe, any microbe, to reliably colonize the infant gut ecosystem. At birth, the infant gut ecosystem is mostly naïve to microbes with open food niches ripe for capture by environmental microbes that enter into the system^106^. Thus, the early colonizers of the gut microbiome, in concert with the food they have access to, determine the microbial constituents that initially persist. This concept is perhaps best exemplified in the seminal study by Jeff Gordon and colleagues who colonized germ-free mice with input “alien” microbial assemblies derived from human, zebrafish, termite, soil, and estuarine environments^107^. All alien microbial assemblies colonized the mouse's gastrointestinal tract with their populations differentially shaped over time by the obvious constraints of that particular host and its diet. Given that such diverse microbial assemblies colonized a mouse gut, it is not hard to image how an infant gut becomes colonized by microbes in its environment. In short, a premature infant gut will be colonized with whatever microbes are available in the neonatal intensive care unit environment and that population will become constrained or amplified by diet, antibiotics, and host effects linked to that particular child^108^. One wonders if this “pioneer” effect is, in part, responsible for the generally beneficial effect seen in meta-analysis of probiotic trials in infants using quite different microbial species (or kingdoms!)^109^. Perhaps, these probiotics simply arrive early via supplementation into the premature infant gut, are shaped by the constraints of that setting, and even though they are “alien” to that environment, they delay arrival and/or establishment of other potentially problematic clades, such as Enterobacteriaceae, Enterococceaceae and Staphylococcaceae.

Two potential desired functions for a probiotic used in premature infants stem from the two observations made by Tissier^3^ more than century ago; dominant growth of bifidobacteria *in situ* is linked to (a) breastfeeding and (b) cognate lowering of the colonic pH. As a mechanistic understanding of the growth of bifidobacteria on HMOs advanced in the last 20 years, an enhanced view of the beneficial role of gut microbiome-generated organic acids, short chain fatty acids (acetate, propionate, and butyrate) and lactic acid produced by the gut microbiome, also emerged^110–113^. In a landmark study, researchers in Japan demonstrated that production of acetate *in situ* by metabolically active bifidobacteria was protective against an otherwise lethal *Escherichia coli* O157:H7 infection^114^. A key discovery was that only strains with the capacity to ferment fructose, the sugar present in the mouse chow, were protective. Indeed, such strains also persisted longer in the mouse intestine, and the authors postulated that increased persistence drove cognate increases in acetate and thus improved protection. For those studying the differential capacity of bifidobacteria to grow on HMOs, the connection to protection of neonates via a similar mechanism was obvious – robust bifidobacterial colonization driven by robust HMO fermentation *should* promote robust protection. But was this hypothesis true in humans? Underwood and colleagues first demonstrated HMO-consuming *BL. infantis* readily colonized premature infants fed breast milk in contrast to *Bifidobacterium animalis* subsp. *lactis*, a strain that lacked HMO consumption capacity^115^. Notably, the *BL. infantis* strain was even detected in infants who were provided the *B. animalis* strain implying cross contamination and/or horizontal transfer by *BL. infantis* into the other infants although a similar transfer did not appear in the reverse direction by *B. animalis*. Subsequent work by the same group showed high-level colonization of healthy breast-fed term infants with supplementation of *BL. infantis* (Evivo^TM^, USA) in comparison to no-probiotic controls^116^. Within days of supplementation, increases in total bifidobacteria in the feces rose from ~20% relative abundance to roughly 80% an increase driven exclusively by the supplemented *BL. infantis*. Once probiotic supplementation ceased on day 28, this high level of *BL. infantis* persisted in the infant microbiome throughout the study suggesting that breastfeeding and its delivery of HMOs was promoting continued, stable, colonization. The fecal biochemistry of the *BL. infantis*-supplemented infants also changed dramatically. Fecal HMOs plummeted with a corresponding rise in fecal organic acids, acetate, and lactate, the end products of *BL. infantis* fermentation. Notably, the average fecal pH of the unsupplemented infants in this trial was 5.97 while that for the supplemented infants was 5.15, a level that mirrors those witnessed in the early 1900s in bifidobacterial-dominated babies of the day^10^. A follow-up study on the same cohort demonstrated the *BL. infantis* strain persisted at a high level in infants with continued breastfeeding for up to 1 year but the strain was less prevalent in subsets of the cohort who received some formula or antibiotic treatments^115^. A recent study demonstrated that premature infants supplemented with *BL. infantis* showed reductions in fecal HMO and increases in fecal organic acids in comparison to a similar cohort supplemented with a non-HMO consuming *Lactobacillus reuteri* strain^117^.

Efforts by Hall and colleagues followed a similar strategy supplementing premature infants with a probiotic cocktail (Infloran, Italy) containing *Lactobacillus acidophilus* and *B. bifidum*^118^. *Lactobacillus acidophilus* is a poor HMO consumer^119^ and as discussed above, *B. bifidum* readily consumes HMOs via an external degradation scheme similar to that employed by *Bacteroides* species^45,120^. Human milk-fed premature infants supplemented in that cohort exhibited higher levels of *B. bifidum* and lower amounts of specific HMO species, namely two fucosyl-lactose isomers, in the feces. In addition, fecal acetate and lactate were higher, and the fecal pH was lower, in the supplemented infants suggesting HMO fermentation by *B. bifidum* colonization. A study by Watkins and colleagues^121^ also employed Infloran and clearly showed colonization by the *Bifidobacterium* genus but did not differentiate species, so it is unclear if an HMO-consuming *B. bifidum* in the Infloran cocktail was indeed a colonizing strain. A recent study employing a different bifidobacterial cocktail (FloraBABY, Renew Life, USA) containing *BL. infantis, B. bifidum, BL. longum*, and *B. breve* strains showed engraftment of all strains in premature infants initially for several weeks after supplementation ceased, however loss of *BL. infantis* occurred by 6 months of age^115,122^. This diversity in responses likely reflects the use of formula in these children (over 50% had some introduction of formula by then) wherein the more breast milk-glycan focused *BL. infantis* was more readily lost as formula is introduced^115^. Surprisingly, even though fecal metabolite analyses were run and differences between the probiotic treated and untreated premature infants were noted, the known fermentation products lactic acid, acetic acid, and 1,2 propanediol (a fermentation product of fucose^41^ that would be expected from bifidobacterial fermentation of HMOs in these children) were not discussed. Recently, Beck and colleagues^123^ employed metagenomic sequencing of a large cohort of preterm infants separately receiving either Infloran (*B. bifidum* and *L. acidophilus*) or Labinic (the U.K.; contains *B. bifidum, BL. infantis* and *L. acidophilus*) and clearly demonstrated that probiotic provision was associated with dramatic changes in the premature infant gut microbiome. Similar to the work by Alcon-Giner^118^ described above, this analysis showed higher persistence of bifidobacteria than *L. acidophilus*. However, only a single unknown metabolite was differentiated from an untargeted metabolomic analysis of stool from probiotic treated and untreated premature infants. Thus, it is unclear if robust HMO fermentation and production of acetic and lactic acids by these input bifidobacterial probiotics was a factor.

While these studies in both term and premature infants demonstrated a high bifidobacterial colonization associated with the supplementation of an HMO-consuming bifidobacterial strain into neonates fed human milk, other factors could have influenced this outcome. Certainly, the pioneer effect, discussed above, likely influenced these outcomes. In addition, human milk contains many antimicrobial compounds including lysozyme^124^, immunoglobins^125^, lactoferrin^126^, antimicrobial peptides^127–129^, glycerol monolaurate,^130^ and lactoperoxidase^131^ among others. Even HMOs can function as anti-infectives by binding to pathogens and deflecting their interaction with the host^132^. Moreover, some milk protein-derived peptides have been shown to directly stimulate bifidobacteria^133,134^. This constellation of anti- and pro-microbial activities likely play a role in shaping the development of infant gut microbiome and facilitating enrichment of bifidobacteria. So how important is HMO-fermentation by key bifidobacteria in the commonly witnessed enrichment of breastfed infants?

Insight into this question has recently emerged. Applications of bovine milk oligosaccharides (that are somewhat similar to HMOs^135^) and a *B. animalis* strain exhibited a synbiotic enrichment *in vitro*, in fermentations of infant feces^136^. Heiss and colleagues^137^ demonstrated that provision of a single HMO (2’-fucosyllactose) along with a cognate 2’-fucosyllactose-consuming *Bifidobacterium pseudocatenulatum* strain^39^ enabled a dramatic five-log increase in *B. pseudocatenulatum* in a wild-type mouse, reaching up to 60% relative abundance in the gut, an increase not witnessed with a different *B. pseudocatenulatum* strain that could not grow on 2’-fucosyllactose. Several aspects of this work are noteworthy. The dramatic enrichment of the *B. pseudocatenulatum* strain both persisted *and increased* after supplementation of the probiotic ceased as long as the 2’-fucosyllactose was provided. Once provision ceased, the supplemented *B. pseudocatenulatum* population rapidly declined. In addition, unlike the naïve infant gut, the adult wild-type mice employed in this study possessed a fully developed microbiome harboring normal colonization resistance^138,139^. Thus, the synbiotic application of HMO plus HMO-consuming bifidobacteria was able to override colonization resistance, indicating that HMO alone can provide an exclusive metabolic niche to the cognate bifidobacterial strain similar to that previously witnessed between porphyrin polysaccharide and select *Bacteroides* strains^140,141^. Another paper recently reinforced this HMO-bifidobacterial synbiotic function, demonstrating that pools of purified HMOs combined with *BL. infantis* promoted engraftment of the strain in both human subjects and gnotobiotic mice containing humanized gut microbiome^142^. Notably, a prior attempt by different authors but with a similar synbiotic combination (pooled HMOs and *BL. infantis*) did not show significant engraftment in mice^143^. While more research is clearly needed in this area, it appears that under some conditions, HMOs alone are capable of enriching cognate HMO-consuming strains in the absence of priority effects or other microbiota modifying factors inherent to breast milk. Importantly, these findings suggest a route to engraft HMO-fermenting bifidobacteria in infants not receiving human milk. The increasing use of pasteurized donor human milk in preterm infants in the NICU is also relevant to this discussion as standard pasteurization techniques utilized by milk banks denature bioactive proteins and peptides, while HMOs are heat-resistant. It is likely that diminished activity of the previously noted human milk components (lactoferrin, immunoglobulins, lactoferrin, antimicrobial peptides, and lactoperoxidase) in donor milk may be partially offset by the abundance of HMOs in donor milk (a pooled product including HMOs from a variety of women). Additional research assessing the impact of individual HMOs, groups of HMOs (e.g. fucosylated vs. sialylated) and single donor vs. pooled donor milk on the gut microbiome and functional outcomes is needed.

## Impact of bifidobacteria on infant health: data from preclinical models and infant biomarker studies

A century ago researchers linked the acid produced in the breast-fed infant colon to preservation of health status and, when absent, its association with disease. As stated by Marriott and Davidson in 1923^12^

*“In a number of instances, it was possible to study the same infants, first while normal, and subsequently when suffering from an infection. There was regularly a marked decrease in gastric acidity in the presence of infection”.*

As discussed above, we now know much more of the mechanism by which bifidobacteria ferment HMOs, producing organic acids, and lowering colonic pH. But does this action improve infant health? Henrick and colleagues^144^ compiled data on the fecal pH of breast-fed infants in studies performed over the last century, noting a clear increase in pH over time and hypothesized different connections to inflammation and immune disorders. Lower fecal pH in infants has been directly linked to reduced stunting^145^ and, recently, morbidity and mortality^146^ in cohorts from less developed regions.

While this reduction in pH is known to come from the production of acetic and lactic acids, other metabolites produced by infant-borne bifidobacteria have been shown to beneficially modulate intestinal function. Indole lactic acid (ILA), a catabolite of tryptophan produced by many intestinal bacteria^147^, including most bifidobacteria^148^, is known to interact with the host aryl hydrocarbon receptors, a key factor in intestinal homeostasis acting on barrier function, epithelial renewal, and various immune cell types^149^. ILA is present at a higher level in feces dominated by bifidobacteria (particularly *BL. infantis*) and is directly anti-inflammatory toward intestinal cells^150–153^. Henrick and colleagues^154^ showed that breastfed infants supplemented with *BL. infantis* had suppressed Th2 and Th17 responses and increased interferon β response compared to no-probiotic controls. Fecal water from the supplemented infants contained high levels of ILA, which in turn upregulated immunostimulatory galectin-1 in Th2 and Th17 cells during polarization. This work provides a nascent mechanistic rationale for why HMO-driven bifidobacterial enrichments in early life may be negatively associated with immune-related diseases^78,155^. More recently, Laursen and colleagues^151^ discovered a larger range of aromatic lactic acids correlated with infant-borne bifidobacterial populations, all linked to a specific aromatic lactic dehydrogenase gene uniquely present in specific infant-borne species—*BL. longum*, *B. breve*, *B. bifidum*, and *BL. infantis*.

Direct demonstrations of the potential health benefits from active, HMO-consuming bifidobacterial probiotics have also emerged in both animal models and human trials. Jena and colleagues^156^ showed that synbiotic applications of *BL. infantis* and bovine milk oligosaccharides reversed nonalcoholic steatohepatitis in a mouse model. Heiss and colleagues^137^ demonstrated high-level colonization by *B. pseudocatenulatum*, driven by simultaneous 2’-fucosyllactose provision, dramatically improved colitis within a mouse model. In both healthy and premature infant cohorts, early colonization by supplemented *BL. infantis* exhibited lowered fecal calprotectin and proinflammatory cytokines^157^ as well as reduced the incidence of antimicrobial resistance genes^117,157–159^.

## Impact of bifidobacteria on infant health: data from clinical studies

Reports on the clinical outcomes of the use of validated, HMO-consuming, bifidobacterial probiotics in premature infants are relatively rare precisely because most probiotic strains employed in the NICU are not assessed for HMO-growth capacity. Indeed, some have speculated that the lack of efficacy in reducing NEC in large clinical trial employing a *B. breve* strain^160^ was due, in part, to the lack of HMO consumption and colonization by the provided strain^161,162^. A number of studies using the probiotic product Infloran containing *L. acidophilus* and either *BL. infantis* or *B. bifidum* (bifidobacterial species that consume HMO) have been shown to reduce the risk of NEC^163–169^. While these studies looked at clinical outcomes, they did not score for bifidobacterial colonization or cognate changes in fecal biochemistries so no direct links to HMO-fermentation can be ascribed. Moreover, analysis of these studies can be confusing as they differentially record the same Infloran product as containing either species *BL. infantis* or *B. bifidum*. Around the same time, our group demonstrated that many probiotic products containing bifidobacterial species (particularly *BL. infantis*) actually had different species in the product than the label indicated^170^. Contamination of NICU-focused commercial probiotics with alternative bifidobacterial species was recently demonstrated by Beck et al.^123^ where a *B. animalis* species, not described on the product label, was observed both directly in the probiotic cocktail and in the supplemented infants. Clearly, a needed component of future NICU probiotic trials is precise validation of input probiotic species.

**Figure 4.**
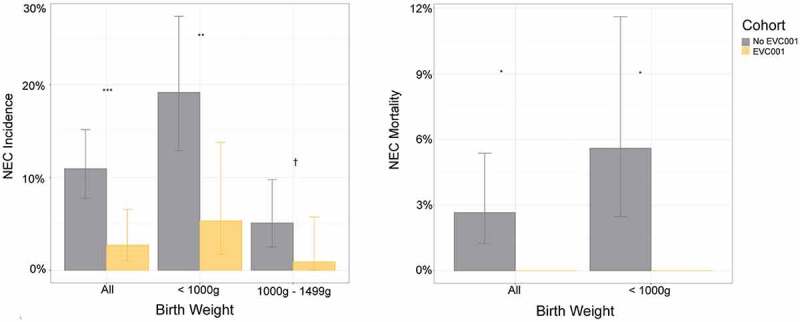
NEC incidence and mortality in the observational study employing *BL. infantis* EVC001 (Evivo^TM^) or no probiotic controls. Left panel, NEC incidence by birth weight and cohort. Error bars show 95% CIs around the estimates. ***P < .001; **P < .01;†*P* < .1, Fisher exact test. Right panel, NEC-related mortality rates by birth weight and cohort. Error bars show 95% CIs around the estimates. *P < .05, Fisher exact test. Reproduced with permission from Tobias et al.^171^.

Recent observational studies employing probiotics in the NICU provide more direct links that the HMO-bifidobacterial axis may be a factor to infant health outcomes. In a companion paper to the study by Alcon-Giner et al.^118^ that demonstrated functional evidence of HMO-fermentation of the input *B. bifidum*, Robertson and colleagues^162^ followed a larger cohort of 982 premature infants over a 10-year period, the latter 5 years of which the infants received a probiotic which changed from a cocktail of *B. bifidum* and *L. acidophilus* (Infloran) to one containing *B. bifidum, BL. infantis*, and *L. acidophilus* (Labinic). As seen in the studies described above, NEC and late-onset sepsis rates were reduced with use of HMO-fermenting bifidobacterial probiotics, however reductions in all-cause mortality were not statistically significant. In a separate study employing a *BL. infantis* probiotic (Evivo^TM^) Tobias and colleagues^171^ demonstrated statistically significant reductions in NEC incidence and NEC-associated mortality in very low birthweight infants (*N* = 483 in cohort), including an extremely low birth weight subgroup (Figure 4). Robust colonization and evidence of HMO fermentation *in situ* for this specific probiotic have been previously validated^115,116,159^. Why might these two recent studies show differences in mortality? First, the overall incidence of NEC was higher in the NICU examined in the Tobias et al.^171^ study, which facilitated a lower overall cohort number needed to reach statistical significance per NEC outcomes. However, additional factors such as notable differences in probiotic species, format (cocktail vs. single strain), formulation (HMO-activated strains vs. not) and dosage may have influenced these outcomes.

Finally, a recent study focused on infants with severe acute malnutrition (SAM) illustrates how a focus on the HMO-fermenting bifidobacteria can help repair growth functions in these at-risk populations as well. Barrett and colleagues^161^ demonstrated that supplementation of *BL. infantis* (Evivo^TM^) in breastfed infants with SAM resulted in significant colonization by the probiotic strain which correlated with improved weight gain and reduced inflammation.

## Summary

Research over the last 20 years has provided key insight into the molecular mechanism of consumption of HMOs by infant-borne bifidobacteria as well as applications of these HMO-consuming strains in the NICU. This new mechanistic understanding of a very old observation – the enrichment of bifidobacteria in breastfed infants – now drives new questions that challenge the concept of a probiotic “function”. While numerous reviews detail the litany of *potential* mechanisms associated with probiotic functions,^172,173^ little research has scored those precise functions for specific host responses and beneficial clinical outcomes in actual human clinical trials employing these probiotics. Indeed, since most probiotics do not robustly colonize the host, it is perhaps not surprising that efficacy in their use across a range of clinical targets is questioned^174^ (see Box 3). We posit that breastfed neonate presents an unusual case where an overt mechanistic link has emerged between select supplemented probiotics and improvement in infant health. The assembled research described herein argues select HMO-consuming bifidobacteria readily colonize the breast-fed infant gut and ferment HMOs to produce organic acids and other key metabolites (such as ILA) which, in turn, beneficially condition the gastrointestinal environment and restrict entry by other, often problematic, microbial taxa. However, numerous questions, research gaps, and debates remain. Several such questions are elaborated below.

Box 3. **The problem with probiotics**.

Since the early proposal as a general concept by Metchnikov^182^ to the development of the first commercial product by Shirota^183^, probiotics have been simultaneously popular and controversial^174,184–186^. The current definition of a probiotic as “Live microorganisms that, when administered in adequate amounts, confer a health benefit on the host” originated in 2001 by FAO/WHO^178^. Many current best-selling probiotics were developed decades ago wherein appropriate commercial concerns (oxygen tolerance, fermentation scale up, strain stability, shelf life) were important considerations for their commercialization in order to deliver a live microbe in “adequate amounts”. Efficacy toward a specific health target was not the sole criteria early on, but rather a more general effort to mitigate “dysbiosis”, typically in the form of increased presence of the probiotic and thus lowering of other pathobiont clades. In general, commercial probiotics have been shown not to persist, much less increase, in the host beyond supplementation and various probiotic trials have not shown significant alterations in the host gut microbiome^187^. This failure of probiotics to contribute quantitatively to the host microbiome is not surprising. Colonization resistance inherent to established gut microbiomes provides protection from exogenous bacterial interlopers, due in part to the established and resilient food networks that form within a health gut ecosystem. Suez and coworkers^174^ proposed that the lack of persistence, level of colonization and associated mucosal interaction is a likely factor impacting efficacy in probiotic trials.

**Question** **– Is a** **greater focus on probiotic *function* in neonates needed?** In terms of the breast-fed infant gut, we argue that microbiota function and its impact on the host should be the chief focus in clinical cohorts assessing the use of probiotics in neonates. Each year brings ever more elaborate microbiome analyses of infant cohorts. Early work on severe acute malnutrition by Jeff Gordon and coworkers employed the use of microbial composition as a measure of “gut microbiota age”, determining a persistent “immaturity” in the gut microbiota of afflicted infants^175^. This concept of using microbial taxa alone to score the chronological age or maturity of the gut microbiome infers a cognate functional component to that microbiome. However, in the absence of a scored microbiota function (pH, organic acids, etc.), how informative is this measurement in neonates? The recent comprehensive metagenomic analysis of probiotic use in the NICU by Beck and colleagues^123^ deemed the probiotic-treated neonates to have experienced an “accelerated maturation” due to the presence of a supplemented bifidobacterial population. However, that same cohort did not witness significant differences in fecal organic acids. Is the mere presence of the supplemented probiotic in the naïve infant gut all that is needed for proper maturation? If so, would continuous delivery of nonviable (i.e. dead) bifidobacterial probiotics have performed the same function? Perhaps, the unusual abundance of HMOs in human breast milk can now be explained by the resulting metabolic output of a robust fermenter-like *BL. infantis* and the functions of those metabolites within the intestine of neonates.

A crude, but useful, analogy emerges from the use of starter cultures in food and beverage fermentations. For centuries, wine and dairy fermentations were carried out wherein the indigenous microbiota (yeast and lactic acid bacteria, respectively) performed the main fermentations. However, advances in fermentation science brought the use of select starter cultures that, upon supplementation, far more reproducibly drove those fermentations. Further sophistication in starter culture science drove novel strain selection as well as strain inoculation and management practices, all for the purpose of maintaining a robust fermentation, a function that was easily measured (e.g., alcohol or acid production or sugar loss). In these industries, a real-time assessment of the fermentation is the norm and any fermentation that lacks proper functional outputs is considered at risk and remediation would start immediately. Now that we better understand the factors behind HMO-driven bifidobacterial colonization of infants, should the NICU adopt a similar approach with premature infants? We argue that there is enough evidence to suggest a path for the NICU to routinely track such fecal fermentation products (e.g. decreased fecal HMOs, increased fecal organic acids, decreased fecal pH) as a means to provide clinicians with critical real-time insight into the nature of the gut fermentation occurring in premature infants. Such routine measurements will similarly provide data the scientific community needs to accurately and quantitatively compare and contrast probiotic trials and ultimately provide an evidential basis to enhance effective treatments.

**Question – What version of probiotic(s) should be used in the NICU?** As described above, there are various probiotic formats containing HMO-consuming strains. Two main themes emerge: (a) use of single strains versus a cocktail of strains and (b) employing strains that catabolize HMO externally (i.e. *B. bifidum* type) vs. internally (i.e. *BL. infantis* type) (as discussed previously, see Figure 2). Strain cocktails are a common format for probiotics. The general rationale for cocktails is obvious – they provide more functions and/or duplications of functions in the face of different host environments. In a sense, this mirrors the concept that multiplicity and diversity of function providing a robust resilience to the adult human gut microbiome to meet a diversity of diets and ecological interfaces (e.g. bacteriocins and bacteriophage), among other interactions. However, the naïve infant gut is a unique environment provided with a unique food, breast milk, which itself is selective for HMO-consuming bacteria, primarily bifidobacteria. Recent work from Katayama and colleagues provides a nascent view of how HMOs might drive different bifidobacterial communities to form^176^. The ability to supplement HMO-consuming bifidobacteria early in life enables neonatologists to direct a bifidobacterial community structure of the early breast-fed infant gut^123^. Choices of internal vs. external HMO degradation formats (Figure 2) will also likely influence community structure as external degradation would be expected to prompt cross-feeding interactions among other gut microbes, be they good^40,44^ or bad^49^. As argued above, perhaps a better rationale for the use of any probiotic is one of function. Which format more reproducibly delivers the most robust HMO-fermentation *in situ*? As this is readily measured, more research on such functional outputs of NICU probiotic use will help decipher this question. Finally, the use of non-HMO consuming species, or postbiotics^177^, in breastfed infants and approaches to optimizing the microbiota of formula-fed infants need more research on possible mechanistic rationales for their efficacy.

## Conclusion

In the last two decades the field of microbiology has experienced an incredible transformation. The advent of “omics” tools, particularly next-generation sequencing to determine uncultured microbial communities has provided a wealth of insight into our gut microbiome and its function. At the same time, general knowledge of probiotics and their use by both physicians and the public has increased dramatically,^178^ yet significant challenges to their clinical efficacy remain^174^. The use of HMO-consuming bifidobacterial probiotics in neonates represents a novel, tailored, use where the direct function of the implanted probiotic is readily observed and can be related to clinical efficacy. We propose that similarly “functional” synbiotic applications^179^ may drive new discoveries, inventions and translations (see Box 4) to advance human health in a range of settings.

Box 4. **The route from discovery to invention to translation**.

*“To him who devotes his life to science nothing can give more happiness than increasing the number of discoveries, but his cup of joy is full when the results of his studies immediately find practical applications.” Louis Pasteur* ^188^

The central goal of research in the life sciences is discovery; how does the world work? Breakthroughs in our understanding of how biology functions from atomic-level mechanisms to ecological behavior of entire communities do not, however, immediately identify how such discoveries can be translated into practice. Invention is the cognitive process of recognizing a *utility* that can be provided by explicitly acting upon new understanding. Scientists, innovators, and regulators alike are faced with major challenges in building utility from the discoveries of microbiome research. Indeed, for the average microbiologist or neonatologist embracing a new discovery, application is not always so easily recognizable. This was the case with the UC Davis Milk Bioactives group (initiated by authors German, Lebrilla and Mills), a multidisciplinary team that examined the HMO-bifidobacterial axis. Early discovery of specific bifidobacteria, most notably *BL. infantis*, consume the bulk of HMOs in mother’s milk^21^ suggested a unique partnership between these abundant oligosaccharides and specific bifidobacterial taxa. The initial excitement of this discovery was the normal academic joy of recognizing a clear and compelling research path to identify the mechanism underlying this phenotype. However, finding a utility for this phenotype was not obvious at the time. It was with the connection to neonatologist (and coauthor) Mark Underwood and witnessing premature infants in his NICU suffering from NEC and infections that the “real world” intersected with our otherwise lofty academic pursuits. The Milk Bioactives group immediately recognized that, *BL. infantis*, the “champion” HMO consumer, should be able to populate the premature infant gut, reduce suffering, and improve outcomes. As a consequence, the company Evolve Biosciences (now Infinant Health) was launched jointly by the University of California, Davis, and the scientists responsible for the discovery research. To date, over 50,000 infants have been supplemented with *BL. infantis* EVC001.

What were the key elements that facilitated this translation? First and foremost, it was the interdisciplinary nature of the UCD Milk Bioactives group. The historic academic model of siloed research can be an anathema to the kind of integrated thinking that advances understanding of complex biology, but it also mutes innovative thinking. A second component was the increasing recognition that translation in the form of entrepreneurial activity is a required (and very much desired) component of a University’s public service. This has not always been the case, particularly at land grant universities where translation was/is typically done in the form of publishing and extension. Thankfully, the fruits of this new thinking are being realized and the field of microbiome science has no shortage of university-fostered startup companies addressing a range of applications.
